# 

**Published:** 1889-05

**Authors:** 


### CONTENTS:

Article I.—The Odontological Society of Great Britain. . 1
" II.—The Dentists of the Day. By Dr. J. B. Patrick. 14
" III.—Short Notes on Diseases of the Palate. By V. A. Latham, B. & C., F. R. M. S. . 20
" IV.—Antiseptics. . . . . . . . . . 24
" V.—The Nervous Patient. By J. R. Callahan, D. D. S. 27
" VI.—On Plastic Fillings. By D. D. Atkinson, D. D. S. 31
" VII.—Application and Uses of the Rubber Dam. By Dr. H. T. Lynch. . . . . . . 34
" VIII—Methyl Chloride for Local Anæsthesia. By Dr. B. Ward Richardson. . . . 38

EDITORIAL.

Dental Law of Colorado. . . . . . . . . . . 40

Chicago Dental Society. . . . . . . . . . . 42

OBITUARY.

Dr. Charles F. Dinger. . . . . . . . . . 43

BIBLIOGRAPHICAL.

Dental Science. . . . . . . . . . . 43
A Text Book of Operative Dentistry. . . . . . . 44
Transactions of the American and Southern Dental Associations. 45
Transactions of New York Odontological Society. . . . 45

MONTHLY SUMMARY.

Washing Amalgam. . . . . . . . . . . . 45

Teeth of Some Animals. . . . . . . . . . . 46

Impression Taking. . . . . . . . . . . . 48

